# The contribution of *Escherichia coli* from human and animal sources to the integron gene pool in coastal waters

**DOI:** 10.3389/fmicb.2014.00419

**Published:** 2014-08-12

**Authors:** Alexandra Moura, Susana Araújo, Marta S. Alves, Isabel Henriques, Anabela Pereira, António C. M. Correia

**Affiliations:** Department of Biology and CESAM, University of AveiroAveiro, Portugal

**Keywords:** environmental reservoirs, microbial risk assessment, multi-resistance, integron diversity, replicon typing, Enterobacteriaceae

## Abstract

To understand the contribution of animal- and human-derived fecal pollution sources in shaping integron prevalence and diversity in beach waters, 414 *Escherichia coli* strains were collected from beach waters (BW, *n* = 166), seagull feces (SF, *n* = 179), and wastewaters (WW, *n* = 69), on the World Biosphere Reserve of the Berlenga Island, Portugal. Statistical differences were found between the prevalence of integrons in BW (21%) and WW (10%), but not between BW and SF (19%). The majority of integrase-positive (*intI*^+^)-strains affiliated to commensal phylogroups B1 (37%), A0 (24%), and A1 (20%). Eighteen different gene cassette arrays were detected, most of them coding for resistances to aminoglycosides, trimethoprim, chloramphenicol, and quaternary ammonia compounds. Common arrays were found among strains from different sources. Multi-resistance to three or more different classes of antibiotics was observed in 89, 82, and 57% of *intI*^+^-strains from BW, SF and WW, respectively. Plasmids were detected in 79% of strains (60/76) revealing a high diversity of replicons in all sources, mostly belonging to IncF (Frep, FIA, and FIB subgroups), IncI1, IncN, IncY, and IncK incompatibility groups. In 20% (15/76) of strains, integrons were successfully mobilized through conjugation to *E. coli* CV601. Results obtained support the existence of a diverse integron pool in the *E. coli* strains from this coastal environment, associated with different resistance traits and plasmid incompatibility groups, mainly shaped by animal fecal pollution inputs. These findings underscore the role of wild life in dissemination of integrons and antibiotic resistance traits in natural environments.

## Introduction

Environmental antibiotic resistance reservoirs are known to represent the origins of the resistance determinants that nowadays constitute major clinical threats (Davies and Davies, 2010; Tacão et al., 2012, 2013; Perry and Wright, 2013). In the recent years much attention has been given to marine environments and migratory birds with increasing evidence of their role in the dissemination of antibiotic resistant Enterobacteriaceae, particularly *Escherichia coli* (Dolejska et al., 2007, 2009; Poeta et al., 2008; Radhouani et al., 2009; Poirel et al., 2012; Hernandez et al., 2013; Kmet et al., 2013; Santos et al., 2013; Veldman et al., 2013). *E. coli* is the predominant facultative anaerobe in gastrointestinal tract of humans and animals (Tenaillon et al., 2010). Although most *E. coli* are commensal, some can be pathogenic and may be transmitted through contaminated water or food, or through contact with animals and people. Pathogenic *E. coli* has been reported as a major cause of mortality as a result of infant diarrhea, extra-intestinal and urinary tract infections, thus constituting an important hospital- and community-acquired pathogen (Guentzel, 1996; Touchon et al., 2009). Due to their genetic flexibility and adaptability to diverse stress conditions, both commensal and pathogenic *E. coli* strains have the ability to persist in terrestrial and aquatic habitats (Van Elsas et al., 2011).

Integrons are bacterial site-specific recombination platforms of acquisition and expression of mobile genes, called gene cassettes (Stokes and Hall, 1989). It has been shown that the persistence of antibiotics in the environment at sub-therapeutic concentrations contributes to the acquisition of antibiotic resistance genes between different strains, mediated by integrons, as a result of the activation of bacterial SOS responses (Baharoglu et al., 2010; Andersson and Hughes, 2011). In addition, integrons are often associated with conjugative plasmids which contribute to their mobilization and wide dissemination (Moura et al., 2012a). The spread of such determinants can constitute serious environmental risks, compromising both ecosystem and human health.

In this study, we aimed to understand the involvement of animal- and human-derived fecal pollution sources in shaping integron prevalence and diversity in beach waters. Sampling was performed in the World Biosphere Reserve of Berlenga Island, located in the Atlantic Ocean, because here the sources of fecal pollution are limited and well-identified, consisting of both animal- and human-derived origins. The Berlenga Island constitutes an important nesting area of sea birds, in particular the yellow-legged gulls (*Larus* [*cachinnans*] *michahellis*), which are, by far, the dominant local fauna and a major source of fecal pollution in the island (Araújo et al., 2014). This island is only circumstantially inhabited by tourists in the summer season, and human-derived wastewaters are discharged near the coastline of the island without prior treatment (Araújo et al., 2014).

To address our aims, we examined the prevalence and diversity of integrons in *E. coli* strains collected from beach waters, as well as from seagull feces and raw wastewaters in the Berlenga Island. The association of integrons and plasmids was also assessed in order to determine the extent of the environmental risk at play.

## Materials and methods

### Sampling, *E. coli* isolation and molecular typing

In a previous study, a collection of 939 *E. coli* isolates was obtained from samples collected between May and September 2011 at the Berlenga Island (Latitude: 39° 24′ 52″ N; Longitude: 9° 30′ 22″ W), located 5.7 miles northwest of Cape Carvoeiro, Portugal. Samples consisted of: (i) beach waters; (ii) composite seagull (*Larus* [*cachinnans*] *michahellis*) fresh fecal samples; and (iii) human-derived raw wastewaters (Araújo et al., 2014). Isolates were selected in Chromocult Coliform Agar, confirmed by plating in MacConkey and mFC agar and 16S rRNA gene sequencing, as previously described (Araújo et al., 2014). Molecular typing was performed by BOX-PCR (Araújo et al., 2014), resulting in a total of 414 different *E. coli* strains that were used in this study.

### Integron screening detection and characterization

*E. coli* strains were screened by PCR for the presence of class 1 and class 2 integrase genes (*intI1* and *intI2*, respectively), as previously described (Moura et al., 2012b). Integrase-positive (*intI*^+^)-strains were further characterized. Class 1 and class 2 integron variable regions were amplified using primers targeting flanking regions of gene cassette arrays (class 1: *intI1* or *attI1* at 5′ region and *tniC, qacE/sul1, or sul3* at 3′ region; class 2: *intI2* or *attI2* at 5′ region and *ybeA* at 3′ region), using the Extensor Long Range PCR Master Mix (Thermo Scientific, USA), as described before (Moura et al., 2012b). Specific primers for gene cassettes were also used in primer walking. All primer sequences are listed in Table 1. Sequences obtained were subjected to BLAST (Altschul et al., 1997) searches against the INTEGRALL database (http://integrall.bio.ua.pt; Moura et al., 2009). Insertion sequences were compared against ISFinder database (http://www-is.biotoul.fr; Siguier et al., 2006) to confirm identity. Gene cassette promoters were annotated according to Jové et al. (2010).

**Table 1:** **Primers used in this study in the characterization of integrons**.

**Primer name^a^**	**Target**	**Sequence (5′–3′)**	**References**
**INTEGRASE GENES**			
intI1F	*intI1*	CCTCCCGCACGATGATC	Kraft et al., 1986
intI1_894F(ER.1.6F)	*intI1*	CCCAGTGGACATAAGCCTG	Moura et al., 2012b
intI1R	*intI1*	TCCACGCATCGTCAGGC	Kraft et al., 1986
intI2F	*intI2*	TTATTGCTGGGATTAGGC	Goldstein et al., 2001
intI2R	*intI2*	ACGGCTACCCTCTGTTATC	Goldstein et al., 2001
**FLANKING REGIONS**			
5′-CS	*attI1*	GGCATCCAAGCAGCAAG	Levesque et al., 1995
3′-CS	3′ conserved segment	AAGCAGACTTGACCTGA	Levesque et al., 1995
qacE-F	*qacE/qacEdelta1*	ATCGCAATAGTTGGCGAAGT	Sandvang et al., 1997
qacE-R	*qacE/qacEdelta1*	CAAGCTTTTGCCCATGAAGC	Sandvang et al., 1997
sul1F	*sul1*	CTGAACGATATCCAAGGATTYCC	Heuer and Smalla, 2007
sul1R	*sul1*	AAAAATCCCATCCCCGGRTC	Heuer and Smalla, 2007
sul3F	*sul3*	AAGAAGCCCATACCCGGRTC	Heuer and Smalla, 2007
sul3R	*sul3*	ATTAATGATATTCAAGGTTTYCC	Heuer and Smalla, 2007
RH506	*tniC*	TTCAGCCGCATAAATGGAG	Post et al., 2007
orf513_6F	IS*CR1*	ATGGTTTCATGCGGGTT	Arduino et al., 2003
orf513_7R	IS*CR1*	CTGAGGGTGTGAGCGAG	Arduino et al., 2003
qnrS_rev2	*qnrS*	CAAATTGGCGCGTAGAGCGCC	This study
hep74	*attI2*	CGGGATCCCGGACGGCATGCACGATTTGTA	White et al., 2001
hep51	*ybeA*	GATGCCATCGCAAGTACGAG	White et al., 2001
**GENE CASSETTE PRIMER WALKING**			
aadA1_F	*aadA1*	TATCAGAGGTAGTTGGCGTCAT	Randall et al., 2004
aadA1_R	*aadA1*	AATGAAACCTTAACGCTATGGAAC	Randall et al., 2004
aacA4F (ER.1.17F)	*aacA4*	CGAGCGAACACGCAGTG	Moura et al., 2012b
dfrA12_F	*dfrA12*	CCCACTCCGTTTATGCGCG	This study
dfrA17_F	*dfrA17*	CACGTTGAAGTCGAAGGTGA	This study
estXF (MM.2.11F)	*sat/estx*	GGCCGAGGATTATCCA	Moura et al., 2007
cmlA_F	*cmlA*	GGACATGTACTTGCCAGCA	This study
cmlA_R	*cmlA*	GGGATTTGAYGTACTTTCCGC	This study
qacH_F	*qacH*	GAGGTCRTCGCAACTTCC	This study
qacH_R	*qacH*	GCGCTGACCTTGGATAGC	This study
linF_F	*linF*	CGCTTGAGGCGGCTGTTTTG	This study
psp_F	*psp*	CCGGATTTTGTGCGGCGGTC	This study
orfF_F	*orfF*	GGCGTTATTCAGTGCCTGTT	This study
IS1_F	IS*1*	CGGTAACCTCGCGCATACAG	This study
ISUnCu_F	IS*UnCu1*	GGACTCTCCCCACAAGTAGTG	This study

a *F, forward; R, reverse*.

### Phylogrouping and antibiotic susceptibility profiles

*E. coli* phylogenetic groups (A0, A1, B1, B2, D1, D2) were determined by PCR using the NZYTaq Green Master Mix (NZYTech, Portugal) and primers and conditions described before (Clermont et al., 2000; Figueira et al., 2011). Antibiotic susceptibilities were tested by disc diffusion agar according to the Clinical and Laboratory Standards Institute recommendations (CLSI, 2012) and using *E. coli* ATCC 25922 as control strain. The following antibiotics were tested: ampicillin (AMP, 10 μg), amoxicillin (AML, 10 μg), amoxicillin + clavulanic acid (AMC, 30 μg), piperacillin (PRL, 100 μg), piperacillin + tazobactam (TZP, 110 μg), cefalothin (CEF, 30 μg), ceftazidime (CAZ, 30 μg), cefotaxime (CTX, 30 μg), gentamicin (GEN, 10 μg), streptomycin (STR, 10 μg), imipenem (IPM, 10 μg), nalidixic acid (NAL, 30 μg), ciprofloxacin (CIP, 5 μg), tetracycline (TET, 30 μg), chloramphenicol (CHL, 30 μg) and trimethoprim/sulfamethoxazole (STX, 25 μg) (Oxoid, Basingstoke, UK).

### Genomic location of integrons and plasmid characterization

To determine the genomic location (plasmid/chromosomal) of integrons, genomic DNA and plasmid DNA were extracted and purified using the Silica Bead DNA Extraction Kit (Thermo Scientific, USA) and the E.Z.N.A. Plasmid Mini Kit II (Omega Bio-tek, GA, USA), respectively. Aliquots were loaded onto 0.9% agarose gels and separated by electrophoresis at 80 V for 80 min. Gels were then stained with ethidium bromide and documented with the Molecular Imager® Gel Doc™ XR System and Image Lab™ Software (Bio-Rad, Hercules, CA, USA). DNA was transferred under vacuum onto positively charged nylon membranes (Hybond N+; Amersham, Freiburg, Germany) and subsequently cross-linked under UV irradiation for 5 min. Hybridizations with *intI1*- and *intI2*-digoxigenin (DIG) labeled probes (Moura et al., 2007, 2012b) were performed overnight in 50% formamide hybridization buffer at 42°C. Detections were carried out using the DIG Nucleic Acid Detection Kit (Roche Diagnostics, Germany) following instructions provided by the manufacturer. Positive and negative controls were included in all experiments to confirm the specificity of detection.

In addition, *intI*^+^-strains were included as donors in mating assays using rifampicin-resistant *E. coli* CV601-GFP (Smalla et al., 2006) as recipient strain, using previously described procedures (Moura et al., 2012a). Briefly, liquid cultures of donor and recipient strains were prepared separately in 10 mL Luria–Bertani broth (LB) without antibiotics and grown overnight with gentle shaking at 28°C. Recipient and donor strains were mixed (ratio 1:1) and centrifuged for 5 min at 6700 g to precipitate cells. Supernatants were discarded and replaced by 1 mL fresh LB. Mixtures were incubated overnight at 28°C without shaking. Cells were then precipitated by centrifugation for 5 min at 6700 g and washed in 0.9% NaCl solution. Serial dilutions were prepared in 0.9% NaCl and aliquots of 100 μL were spread on Plate Count Agar plates supplemented with rifampicin (50 mg.L^−1^) and streptomycin (50 mg.L^−1^). Putative transconjugants were grown at 28°C for 48 h. Assays were run in duplicate. Donor and recipient were also placed on the selective plates for mutant detection. Putative transconjugants growing in plates were confirmed by BOX-PCR typing by comparison with donor and recipient banding profiles. BOX-PCR reaction mixtures of 25 μL consisted of 0.5 × NZYTaq Green Master Mix (NZYtech, Portugal), 0.8 μM of primer BOXAIR (5′-CTACGGCAAGGCGACGCTGACG-3′; Versalovic et al., 1991) and 1 μL of cell suspension prepared in 100 μL of distilled water (~1.0 McFarland turbidity standard). Amplification was carried out as follows: initial denaturation for 7 min at 94°C, then 30 cycles of denaturation at 94°C for 1 min, followed by annealing at 53°C for 1 min and extension at 65°C for 8 min, and a final extension at 65°C for 16 min. Generated profiles were separated in 1.5% agarose gels in TAE buffer 5× (50 mM Tris, 50 mM boric acid, 0.5 mM EDTA), at 50 V for 95 min, and stained with ethidium bromide. Plasmid DNA from transconjugants were extracted using E.Z.N.A. Plasmid Mini Kit II (Omega Bio-Tek, Georgia, USA), according to the manufacturer's instructions. Among transconjugants, diversity of plasmids was evaluated by PstI/Bst1770I restriction analyses and replicon typing, as previously described (Carattoli et al., 2005; Moura et al., 2012a). The antibiotic susceptibilities patterns of transconjugants were determined by the disc diffusion method as described above.

### Statistical analyses

Pearson Chi-squared test (χ^2^) was used to test the statistical significance (*P*) of the distribution of integrons and replicons in the different sample sources. Associations were considered significant when *P* was <0.05.

### Nucleotide sequence accession numbers

All integron sequences determined in this study were deposited in GenBank under the accession numbers KF921520 to KF921601.

## Results and discussion

In this study, we investigated the occurrence of integrons and associated plasmids in *E. coli* strains (*N* = 414) from the World Biosphere Reserve of the Berlenga Island. Our goal was to understand whether the source of pollution, i.e., seagull feces (SF) and human-derived wastewaters (WW), influenced integron prevalence and diversity in *E. coli* from beach waters (BW).

**Figure 1:**
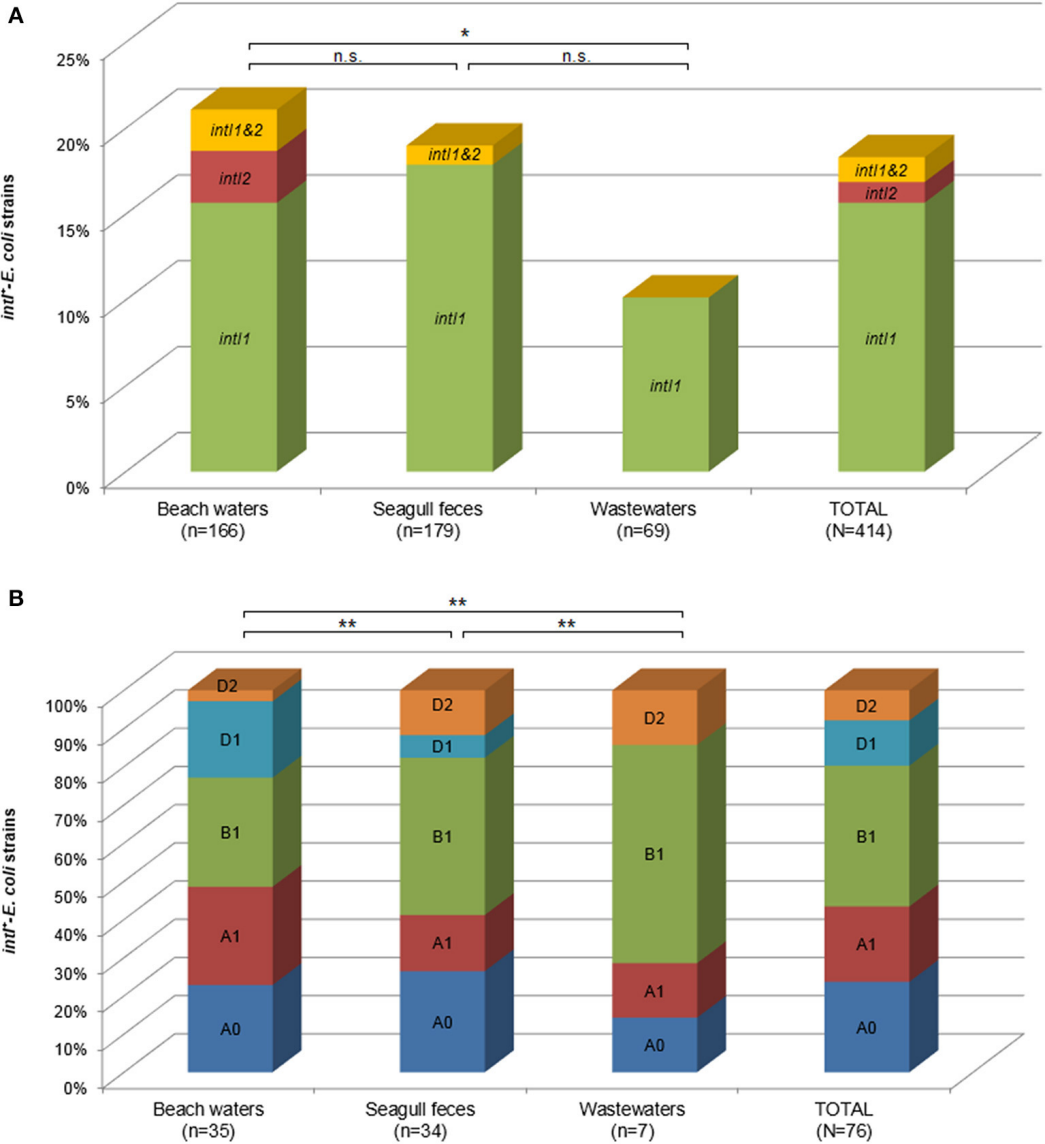
**Prevalence of *intI^+^-E. coli* detected in the Berlenga Island among different sources (A) and phylogroups (B)**. Statistical significance: ^*^P < 0.05; ^**^P < 0.01; n.s., not significant.

Overall, nearly 20% (76/414) of strains harbored *intI* genes (Figure 1A). Prevalence of class 1 and class 2 integron integrases was 18 and 2% in BW, 19 and 0.5% in SF and 10 and 0% in WW, respectively. Previous studies targeting antibiotic resistant bacteria in similar environments (Dolejska et al., 2009) have reported comparable prevalence of class 1 integrons in *E. coli* from surface waters (21%) and black-headed gulls (*Larus ridibundus*) nesting nearby (15%), although with higher prevalence of *intI2* in gulls (11%). Prevalence found at the untreated effluent of Berlengas was also similar to those found in raw human- and animal-derived wastewaters (Moura et al., 2007, 2012b). In this study, differences between prevalence of *intI* genes in BW and WW were statistically different (χ^2^_1_ = 3.98; *P* < 0.05), but not between BW and SF (χ^2^_1_ = 0.261; *P* > 0.05). These results confirm the significant contribution of seagull microbiota in shaping the prevalence of integrons in this ecosystem.

Phylotyping showed a wide intraspecific diversity among integron carrying (*intI*^+^)-*E. coli*. As shown in Figure 1B, the majority of *intI*^+^-strains affiliated to commensal phylogroups B1 (37%), A0 (24%), and A1 (20%). The prevalence of *intI* genes among phylogroups was not statistically significant (χ^2^_5_ = 4.70; *P* > 0.05), being more constraint by the association of the different *E. coli* phylogroups to the different ecological niches (Figure 1B).

Table 2 provides the detailed characterization of the 76 *intI^+^-E. coli* strains obtained in this study. Up to 18 different gene cassettes were found organized into 18 distinct arrays (summarized in Table 3). Common arrays were found among strains from different sources. Gene cassettes detected coded for resistance to aminoglycosides (*aadA1*, Δ*aadA1, aadA2, aadA5, aadB, aacA4, sat2*), trimethoprim (*dfrA1, dfrA12, dfrA14, dfrA17*), chloramphenicol (*cmlA1, catB3*), lincosamides (*linF*) and quaternary ammonia compounds (*qacH*). In addition, gene cassettes coding for putative esterases (*estX*) and phosphoserine phosphatases (*psp*), as well gene cassettes of unknown function (*orfF*) were also present. Though not as part of gene cassettes, genes coding for quinolone resistance (*qnrS1*), quaternary ammonia compounds (*qacEdelta1*) and sulfonamides (*sul1, sul3*) were also associated with the integrons found.

**Table 2:** **Characteristics of *E. coli intI*^+^-strains detected in this study**.

**Strain^a^**	**Phylogroup**	**Antibiotic resistance (and Intermediary) phenotype^b^**	**pDNA replicons^c^**	***intI1***	***intI2***	**Pc promoter^d^**	**Class 1 integron**	**Pc2 promoter^e^**	**Class 2 integron**	**Conjugation^f^**	**Location^g^**	**Accession no(s)**.
F38	A1	STR, TET	n.d.	+	–	PcW-P2	*intI1-aadA1*-3CS		–		C	KF921520
F109	B1	AMP, AML, PRL, NAL, CIP, TET, STX	Frep, FIB	+	–	PcW	*intI1-aadA1*-3CS		–		C	KF921521
F120	B1	STR, TET, STX	Frep, K	+	–	PcW-P2	*intI1-aadA1*-3CS		–		C	KF921522
F202	D2	GEN, STR, TET	Frep, FIB, I1	+	–	PcW-P2	*intI1-aadA1*-3CS		–		C	KF921523
A4	A0	**AMP**, (AMC), **AML**, (CEF), **PRL**, **STR**, TET, **CHL**, **STX**	Frep	+	+	PcS	*intI1-dfrA12-tniC*	n.d.	*intI2-estX-sat2-aadA1-yebA*	+ [Frep]	P	KF921524; KF921591
A9	A1	AMP, (AMC), AML, PRL, (CAZ), CEF, CTX, STR, NAL, CIP, TET, (CHL), STX	n.d.	+	–	PcW_TGN-10_	*intI1-dfrA12-tniC*		–		P	KF921525
A85	A1	AMP, AML, PRL, (CEF), GEN, STR, NAL, CIP, TET, STX	Frep, FIB, I1	+	–	PcW_TGN-10_	*intI1-dfrA12-tniC*		–		C	KF921526
A237	A0	AMP, (AMC), AML, PRL, CEF, CTX, (STR), NAL, TET, CHL, STX	Frep	+	–	PcW_TGN-10_	*intI1-dfrA12-tniC*		–		C	KF921527
A300	A1	AMP, AML, PRL, (CEF), STR, CHL, STX	Frep, FIB	+	–	PcS	*intI1-dfrA12-tniC*		–		C	KF921528
F31	A0	(AMP), (AML), (CEF), STR, TET, STX	n.d.	+	–	PcW-P2	*intI1-dfrA12-tniC*		–		C	KF921529
F33	A0	NAL, (STR), TET, STX	n.d.	+	–	PcW-P2	*intI1-dfrA12-tniC*		–		C	KF921530
F63	A0	AMP, AML, (AMC), PRL, CEF, STR, TET, CHL, STX	n.d.	+	–	PcW_TGN-10_	*intI1-dfrA12-tniC*		–		P	KF921531
F180	B1	STR, CHL, STX	Frep, N	+	–	PcH1	*intI1-dfrA12-tniC*		–		P	KF921532
E109	B1	AMP, AML, AMC, PRL, (CEF), (STR), TET, (CHL), STX	n.d.	+	–	PcW_TGN-10_	*intI1-dfrA12-tniC*		–		P	KF921533
E137	A1	AMP, AML, AMC, PRL, IPM, STR, TET, CHL, STX	I1	+	–	PcW_TGN-10_	*intI1-dfrA12-tniC*		–		C	KF921534
F123	B1	AMP, AML, PRL, TET, STX	Y	+	–	PcH1	*intI1-dfrA14*-IS2*6*		–		C	KF921535
F192	B1	STR, STX	n.d.	+	–	PcW	*intI1-dfrA17*-3CS		–		C	KF921536
A57	A0	AMP, AML, (AMC), (PRL), CEF, GEN, STR, NAL, CIP, TET, STX	FIA, FIB	+	–	PcH1	*intI1-dfrA17*-IS*1*		–		P	KF921537
F29	B1	AMP, AML, AMC, PRL, (CEF), NAL, CIP, (STR), TET, CHL, (STX)	Frep, FIB	+	–	PcH1	*intI1-dfrA17*-IS*26*		–		C	KF921538
A7	A1	**AMP**, (AMC), **AML**, (CAZ), PRL, CEF, CTX, **STR**, NAL, CIP, **TET**, (**CHL**), **STX**	Frep, FIB, I1	+	–	PcW	*intI1-dfrA1-aadA1*-3CS		–	+ [Frep, FIB]	P	KF921539
A25	A1	AMP, AML, (PRL), (CEF), STR, TET, STX	n.d.	+	–	PcW	*intI1-dfrA1-aadA1*-3CS		–		C	KF921540
A47	A1	AMP, AML, (PRL), STR, TET, STX	Frep	+	–	PcW	*intI1-dfrA1-aadA1*-3CS		–		C	KF921541
A62	A1	AMP, AML, PRL, (CEF), STR, NAL, TET, CHL, STX	Frep	+	–	PcW	*intI1-dfrA1-aadA1*-3CS		–		C	KF921542
A94	A1	AMP, AML, (PRL), (CEF), STR, TET, CHL, STX	Frep	+	–	PcW	*intI1-dfrA1-aadA1*-3CS		–		C	KF921543
A102	D1	**AMP**, **AML**, (**PRL**), **STR**, CIP, **TET**, **CHL**	Frep, FIB	+	–	PcW	*intI1-dfrA1-aadA1*-3CS		–	+ [Frep, FIB]	P	KF921544
A154	B1	AMP, AML, PRL, (CEF), STR, TET, STX	n.d.	+	–	PcW	*intI1-dfrA1-aadA1*-3CS		–		C	KF921545
F11	D1	AMP, AML, PRL, STR, TET, CHL, STX	Frep, FIB	+	–	PcW	*intI1-dfrA1-aadA1*-3CS		–		C	KF921546
F17	D1	AMP, AML, PRL, (CEF), STR, TET, STX	Frep, FIB	+	–	PcW	*intI1-dfrA1-aadA1*-3CS		–		C	KF921547
F65	B1	**AMP**, (AMC), **AML**, **PRL**, (CEF), **STR**, NAL, CIP, **TET**, **STX**	Frep, FIB	+	–	PcW	*intI1-dfrA1-aadA1*-3CS		–	+ [Frep, FIB]	P	KF921548
F351	B1	**AMP**, (AMC), **AML**, (**PRL**), **STR**, NAL, CIP, **TET**, **CHL**, **STX**	Frep, FIB, I1	+	–	PcW	*intI1-dfrA1-aadA1*-3CS		–	+ [Frep, FIB]	P	KF921549
F358	B1	**AMP**, ([AMC]), **AML**, PRL, (**CEF**), **STR**, **TET**, **STX**	Frep, FIB, I1	+	–	PcW	*intI1-dfrA1-aadA1*-3CS		–	+ [Frep, FIB, I1]	P	KF921550
A108	B1	AMP, AML, STR, NAL, CIP, TET, CHL, STX	Frep	+	–	PcH1	*intI1-dfrA17-aadA5*-3CS		–		P	KF921551
A180	B1	**AMP**, **AML**, (**PRL**), **STR**, NAL, **TET**, **CHL**, **STX**	Frep, FIB	+	–	PcH1	*intI1-dfrA17-aadA5*-3CS		–	+ [Frep, FIB]	P	KF921552
E108	B1	**AMP**, **AML**, **AMC**, **PRL**, ([CEF]), IPM, **STR**, NAL, CIP, **TET**, **CHL**, **STX**	Frep, FIB	+	–	PcS	*intI1-aacA4-catB3-dfrA1*-3CS		–	+ [Frep, FIB]	P	KF921553
F255	A1	AMP, AML, PRL, GEN, STR, NAL, TET, CHL, STX	Frep, FIB	+	–	PcW-P2	*intI1-aadB-aadA1*-IS*UnCu1*-3CS		–		C	KF921554
E80	B1	**STR**, (NAL), (CIP), CHL	I1	+	–	PcH1	*intI1-aadA2-linF*-IS*26*-IS*Kp19*(trunc.)*-qnrS1*		–	+ [I1]	P	KF921555
A172	D1	AMP, (AMC), AML, PRL, CEF, GEN, STR, NAL, CIP, TET, CHL, STX	Frep	+	–	PcH1	*intI1-estX-psp-aadA2-qacH*-IS*440-sul3*		–		C	KF921556
A33	A0	AMP, (AMC), AML, (PRL), (IPM), STR, TET, CHL, STX	I1	+	+	PcH1	*intI1-estX-psp-aadA2-cmlA1-aadA1-qacH*-IS*440-sul3*	Pc2A	*intI2-dfrA1-sat2-aadA1-yebA*		C	KF921557; KF921594
A107	D1	AMP, AML, PRL, STR, TET, CHL	Frep, FIB	+	–	PcH1	*intI1-estX-psp-aadA2-cmlA1-aadA1-qacH*-IS*440-sul3*		–		C	KF921558
A110	A0	(STR), (NAL), (CIP)	Frep	+	+	PcW	*intI1-estX-psp-aadA2-cmlA1-aadA1-qacH*-IS*440-sul3*	n.d.	*intI2-dfrA1-sat2-aadA1-yebA*		C	KF921559; KF921597
A127	D1	AMP, AML, PRL, STR, CIP, TET, CHL	Frep, FIB	+	–	PcH1	*intI1-estX-psp-aadA2-cmlA1-aadA1-qacH*-IS*440-sul3*		–		C	KF921560
A128	D1	AMP, AML, PRL, (CEF), STR, NAL, CIP, TET, STX	FIB	+	–	PcH1	*intI1-estX-psp-aadA2-cmlA1-aadA1-qacH*-IS*440-sul3*		–		P	KF921561
A148	D1	**AMP**, **AML**, **PRL**, **STR**, **TET**, **CHL**	Frep, FIB	+	–	PcH1	*intI1-estX-psp-aadA2-cmlA1-aadA1-qacH*-IS*440-sul3*		–	+ [Frep, FIB]	P	KF921562
A176	D2	STR, CHL	Frep, I1	+	–	PcW	*intI1-estX-psp-aadA2-cmlA1-aadA1-qacH*-IS*440-sul3*		–		C	KF921563
A182	B1	AMP, AML, PRL, (CEF), STR, TET, (CHL)	Frep, I1	+	–	PcH1	*intI1-estX-psp-aadA2-cmlA1-aadA1-qacH*-IS*440-sul3*	n.d.	–		C	KF921564
F18	A0	AMP, AML, PRL, (CEF), STR, TET, CHL, STX	n.d.	+	+	PcH1	*intI1-estX-psp-aadA2-cmlA1-aadA1-qacH*-IS*440-sul3*	n.d.	*intI2-dfrA1-sat2-aadA1-yebA*		C	KF921565; KF921600
F317	A0	AMP, AML, PRL, (NAL), TET, CHL	Frep	+	–	PcW	*intI1-estX-psp-aadA2-cmlA1-aadA1-qacH*-IS*440-sul3*		–		C	KF921566
F368	A0	AMP, AML, (PRL), (NAL), (CIP), STR, TET, (CHL), STX	n.d.	+	–	PcH1	*intI1-estX-psp-aadA2-cmlA1-aadA1-qacH*-IS*440-sul3*		–		C	KF921567
F380	B1	AMP, (AMC), AML, PRL, CEF, NAL, CIP, (STR), TET, CHL, STX	Frep	+	–	PcW	*intI1-estX-psp-aadA2-cmlA1-aadA1-qacH*-IS*440-sul3*		–		C	KF921568
E3	B1	(STR), TET	Frep	+	–	PcH1	*intI1-estX-psp-aadA2-cmlA1-aadA1-qacH*-IS*440-sul3*		–		C	KF921569
A30	A0	AMP, AML, (IPM), GEN, STR, TET, CHL, STX	Frep, I1	+	+	PcS	*intI1-dfrA12-orfF-aadA2-cmlA1-aadA1-qacH*-IS*440-sul3*	n.d.	*intI2-estX-sat2-aadA1-yebA*		C	KF921570; KF921593
A49	A1	CEF, STR, TET, CHL, STX	Frep	+	–	PcW_TGN-10_	*intI1-dfrA12-orfF-aadA2-cmlA1-aadA1-qacH*-IS*440-sul3*		–		C	KF921571
F278	A1	AMP, AML, PRL, STR, TET, CHL, STX	Frep, FIB, I1, N	+	+	PcW_TGN-10_	*intI1-dfrA12-orfF-aadA2-cmlA-1aadA1-qacH*-IS*440*-IS*10-sul3*	Pc2A-Pc2B	*intI2-dfrA1-sat2-aadA1-yebA*		C	KF921572; KF921601
A115	D1	(STR), TET	Frep, I1	+	–	PcW	*intI1*		–		C	KF921573
A222	B1	AMP, AML, PRL, (CEF), STR, NAL, CIP, STX	Frep, I1	+	–	PcH1	*intI1*		–		P	KF921574
A270	B1	**AMP**, **AML**, **PRL**, (CEF), **STR**, NAL, CIP, **TET**, **CHL**, **STX**	Frep, FIB	+	–	PcH1	*intI1*		–	+ [Frep, FIB]	P	KF921575
A280	B1	STR, TET, STX	Frep, I1	+	–	PcH1	*intI1*		–		C	KF921576
F20	B1	AMP, AML, PRL, (CEF), STR, (CIP), TET, CHL, STX	Frep, FIB	+	–	PcW	*intI1*		–		C	KF921577
F42	A1	AMP, AML, PRL, (CEF), STR, NAL, CIP, TET, CHL (STX)	Frep, I1	+	–	PcH1	*intI1*		–		C	KF921578
F59	B1	**AMP**, [AMC], **AML**, **PRL**, ([CEF]), (IPM), **STR**, **TET**, **STX**	Frep, FIB	+	–	PcW	*intI1*		–	+ [Frep, FIB]	P	KF921579
F84	A1	AMP, AML, (PRL), (CEF), (STR), (NAL), TET	n.d.	+	–	PcW	*intI1*		–		C	KF921580
F140	B1	STR, NAL, CIP, STX	Frep, FIB	+	–	PcH1	*intI1*		–		C	KF921581
F153	A0	AMP, AML, PRL, (CEF), (STR), TET, STX	Frep	+	–	PcH1	*intI1*		–		C	KF921582
F154	B1	AMP, AML, PRL, **STR**, NAL, CIP, **STX**	Frep	+	–	PcH1	*intI1*		–	+ [Frep]	P	KF921583
F158	D2	**AMP**, **AML**, **PRL**, (**CEF**), **STR**, **TET**, **STX**	FIB	+	–	PcW	*intI1*		–	+ [FIB]	P	KF921584
F217	D2	GEN, STR, TET	Frep, I1	+	–	PcW-P2	*intI1*		–		C	KF921585
F354	A0	TET, STX	n.d.	+	–	PcH1	*intI1*		–		C	KF921586
F355	A0	([AMP]), ([AML]), [CEF], **GEN**, **STR**, **TET**, **CHL**	I1	+	–	PcW_TGN-10_	*intI1*		–	+ [I1]	P	KF921587
F357	D2	AMP, AML, (PRL), STR, TET, STX	Frep	+	–	PcW	*intI1*		–		C	KF921588
E18	D2	AMP, AML, PRL, (IPM), STR, CIP, TET, STX	FIB	+	–	PcW	*intI1*		–		C	KF921589
E77	A0	(STR)	n.d.	+	–	PcH1	*intI1*		–		C	KF921590
A6	A0	AMP, AML, AMC, PRL, (CEF), STR, TET, CHL	n.d.	–	+	–	–	n.d.	*intI2-estX-sat2-aadA1-yebA*		C	KF921592
A93	A0	TET, (STR), STX	n.d.	–	+	–	–	n.d.	*intI2-dfrA1-sat2-ΔaadA1-yebA*		C	KF921595
A109	B1	STR, NAL, TET	Frep, FIB	–	+	–	–	n.d.	*intI2-dfrA1-sat2-aadA1-yebA*		C	KF921596
A113	B1	(IPM), STR, NAL, TET	Frep	–	+	–	–	n.d.	*intI2-dfrA1-sat2-aadA1-yebA*		C	KF921598
A142	B1	STR, NAL, TET	Frep	–	+	–	–	n.d.	*intI2-dfrA1-sat2-aadA1-yebA*		C	KF921599

a *Strains A#, F#, and E# were obtained from beach waters, seagull feces and raw wastewaters, respectively*.

b *AMP, ampicillin; AML, amoxicillin; AMC, amoxicillin + clavulanic acid; PRL, piperacillin; CEF, cefalothin; CAZ, ceftazidime; CTX, cefotaxime; GEN, gentamicin; STR, streptomycin; IPM, imipenem; NAL, nalidixic acid; CIP, ciprofloxacin; TET, tetracycline; CHL, chloramphenicol; and STX, trimethoprim/sulfamethoxazole.Intermediary resistance phenotypes are shown in brackets. Phenotypes shown by donors and transconjugants are highlighted in bold. Phenotypes observed in transconjugants but not in donors are shown in square brackets*.

c *Plasmid incompatibility groups determined by replicon typing in E. coli donor strains; n.d., not detected*.

d *Class 1 promoter variants: PcS, TTGACA-N17-TAAACT; PcW, TGGACA-N17-TAAGCT; PcH1, TGGACA-N17-TAAACT; PcH2, TTGACA-N17-TAAGCT; P2, TTGTTA-N17-TACAGT (Jové et al., 2010)*.

e *Class 2 promoter variants: Pc2A, TTTTAA-N17-TAAAAT; Pc2B, TTGTAT-N16-TTTAAT (Jové et al., 2010)*.

f *Transfer of intI-conjugative plasmids in mating assays; replicon types detected in intI-transconjugants are shown in square brackets*.

g *Genomic location, as determined by mating assays and hybridization of plasmid and genomic DNA using intI probes: P, plasmid; C, chromosome*.

**Table 3:**
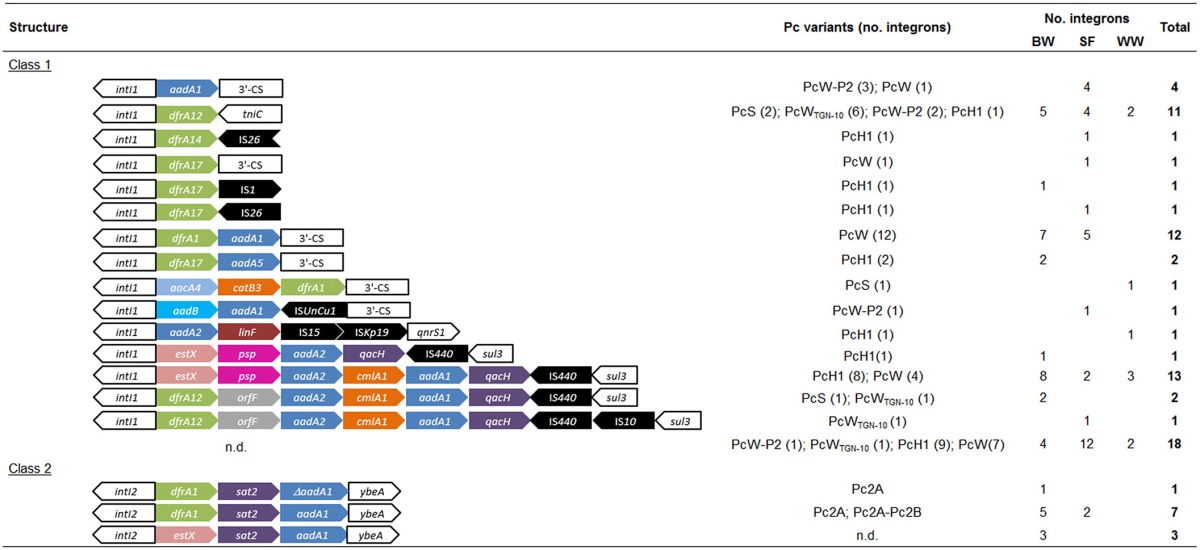
**Overview of the gene cassette arrays and Pc promoter variants present in the 82 integron structures detected in this study among *intI*^+^-*E. coli* strains isolated from beach waters (BW), seagull feces (SF) and wastewaters (WW)**.

**Figure 2:**
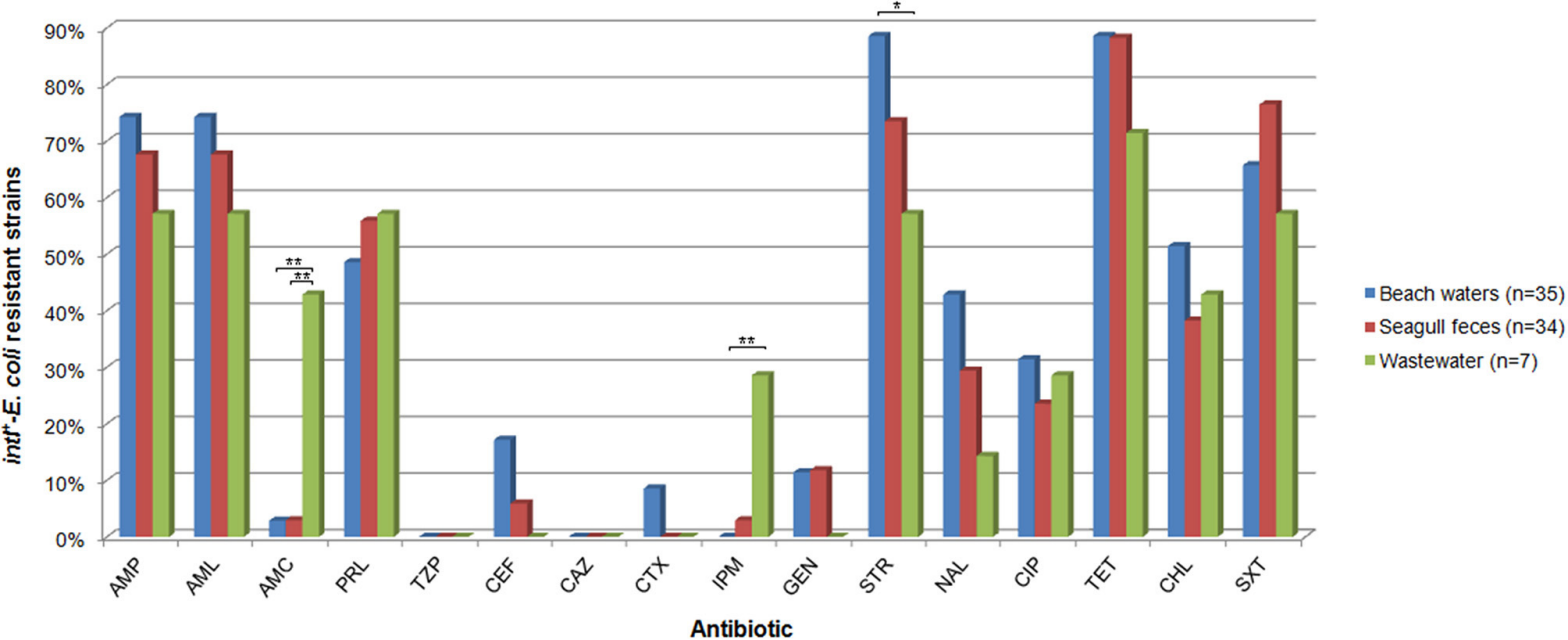
**Prevalence of antimicrobial resistance in *intI^+^-E. coli* strains**. Antibiotic abbreviations: AMP, ampicillin; AML, amoxicillin; AMC, amoxicillin + clavulanic acid; PRL, piperacillin; TZP, piperacillin + tazobactam; CEF, cefalothin; CAZ, ceftazidime; CTX, cefotaxime; GEN, gentamicin; STR, streptomycin; IPM, imipenem; NAL, nalidixic acid; CIP, ciprofloxacin; TET, tetracycline; CHL, chloramphenicol; STX, trimethoprim/sulfamethoxazole. Only statistical significant differences are shown: ^*^P < 0.05; ^**^P < 0.01.

Thus, integron structures detected contained genes involved in diverse resistance mechanisms, including enzymatic antibiotic modification (*aadA, aadB, aacA, catB, sat, sul*), efflux pumps (*qacH, qacE*) and target protection proteins (*qnrS*). This diversity of resistance mechanisms largely contributed to the high prevalence of multiresistant *intI*^+^-*E. coli* (64/76, 83%; considering simultaneous resistance to 3 or more different classes of antibiotics), although the presence of additional mechanisms of resistance besides those within integrons cannot be excluded. Prevalence of multi-resistant strains in BW (89%) was statistically different from that observed in WW (57%), but not to the one observed in SF (82%). Overall, the most frequently resistances detected were against tetracycline (87%), streptomycin (79%), ampicillin (70%), amoxicillin (70%), trimethoprim-sulfamethoxazole (70%), piperacillin (53%), and chloramphenicol (45%) (Figure 2). Differences among sources were not statistically significant, except for resistances against amoxicillin+clavulanic acid and imipenem, that were more prevalent in wastewaters (*P* < 0.01). The prevalence and risk of dissemination of resistant strains to last-resort antibiotics, such as imipenem, is nowadays a matter of great concern, reducing treatment options for infectious diseases. Imipenem resistance if often associated to the presence of integron-borne carbapenemase gene cassetes, such as *bla*_VIM_, *bla*_IMP_, and *bla*_GES_ (INTEGRALL database, Moura et al., 2009) and/or plasmid-borne carbapenemases, such as *bla*_KPC_, *bla*_OXA−48_, and *bla*_NDM−1_ (Carattoli, 2013). Nevertheless, none of these mechanisms have been detected in these strains (Alves et al., 2014). Further investigations will allow to elucidate the mechanisms of carbapenem resistance present in these strains as well their potential risk of dissemination into natural environments.

Different insertion sequences (IS*1*, IS*10*, IS*15*, IS*26*, IS*440*, IS*Kp19*, IS*UnCu1*) were also found within 50% (9/18) of the different arrays (Tables 2–3). Comparative analyses of 20 *E. coli* genomes have also shown the presence of a large number of IS-like elements, constituting 21% of all genes annotated (Touchon et al., 2009) and likely to contribute to the high genome dynamics and adaptation seen in *E. coli*.

Class 1 integrons lacking the 3′-conserved segment (*qacEdelta1/sul1*) represented nearly half of *int1I*^+^-*E. coli* strains (33/71; 46.5%). These included *sul3*-type (*n* = 17) integrons and Tn*402*-derivative integrons containing *tniC* (*n* = 11). Dissemination of *sul3*-containing elements linked to class 1 integrons with an unusual 3′CS region has been reported among clinical and meat-associated *Salmonella* and *E. coli* isolates, including in poultry, often as large platforms with the structures *intI1*-*dfrA12-orfF-aadA2-cmlA1-aadA1-qacH*-IS*440-sul3* or *intI1*-*estX-psp-aadA2-cmlA1-aadA1-qacH*-IS*440-sul3* (Antunes et al., 2007; Sáenz et al., 2010; Curiao et al., 2011; Pérez-Moreno et al., 2013), as observed in this study. Although some variations in these array structures may occur, such as additional IS insertions (e.g., IS*10* in *intI1*-*dfrA12-orfF-aadA2-cmlA1-aadA1-qacH*-IS*440*-IS*10-sul3*, Tables 2–3) or gene cassette deletions (e.g., *cmlA1-aadA1* in the structure *intI1*-*estX-psp-aadA2-qacH*-IS*440-sul3*, Tables 2–3), the apparent conservation and dissemination of these arrays among isolates from different sources and countries, suggest their mobilization through horizontal gene transfer or specific clone dissemination and diversification, rather than cumulative gene cassette acquisition.

Tn*402*-derivative integrons are thought to be the progenitors of classical class 1 integrons that contain the 3′-conserved segment (Post et al., 2007). Integrons derived from Tn*402* are flanked by the *tniC* gene (also called *tniR*) that makes part of the transposition *tniABQC* module. Reports of *tniC*-like integrons are scarce likely because gene cassette characterization usually relies only on the amplification of 3′-CS conservative region (Post et al., 2007). At INTEGRALL database, *tniC*-integrons have been identified in few *Pseudomonas putida, Pseudomonas aeruginosa, Aeromonas caviae* and IncP-1 plasmids, many of those containing gene cassettes coding resistance against beta-lactams (*bla*_VIM_, *bla*_OXA_, *bla*_NPS−1_), aminoglycosides (*aacA4, aacA7, aacC5, aadA1, aadA11*) and trimetophrim (*dfrB5*). In this study, all *tniC*-integrons carried the dihydrofolate reductase *dfrA12* gene cassette, coding for resistance to trimethoprim, and it constitutes the first report on *tniC*-like integrons in *E. coli*.

No significant differences were found on promoter distribution accordingly to sample origin (χ^2^_10_ = 16.25; *P* > 0.05), contrarily to what has been observed in animal- and human-derived wastewaters (Moura et al., 2012c). The majority of integrons detected possessed weak Pc promoter variants (PcW and PcH), which are known to be associated to weak expression of gene cassette arrays (Jové et al., 2010). PcH1 and PcW variants were more prevalent among A0 and B1 phylogroups (χ^2^_20_ = 36.56; *P* < 0.01). Previous studies concerning aquatic environments have also reported higher prevalence of weaker promoters among environmental strains (Moura et al., 2012c; Tacão et al., 2014), as well as studies concerning commensal microbiota (Soufi et al., 2009). Weaker Pc variants are associated to a higher capacity for gene cassette rearrangements, leading to more dynamic arrays (Jové et al., 2010). Interestingly, among *tniC*-type integrons stronger Pc variants were identified: PcS (*n* = 2), PcW_TGG−10_ (*n* = 6), PcW-P2 (*n* = 1). These results corroborate that integron platforms had probably evolved to favor high rate of gene cassette recombination compensating low expression levels and contributing to genome plasticity, as discussed before (Moura et al., 2012c).

**Figure 3:**
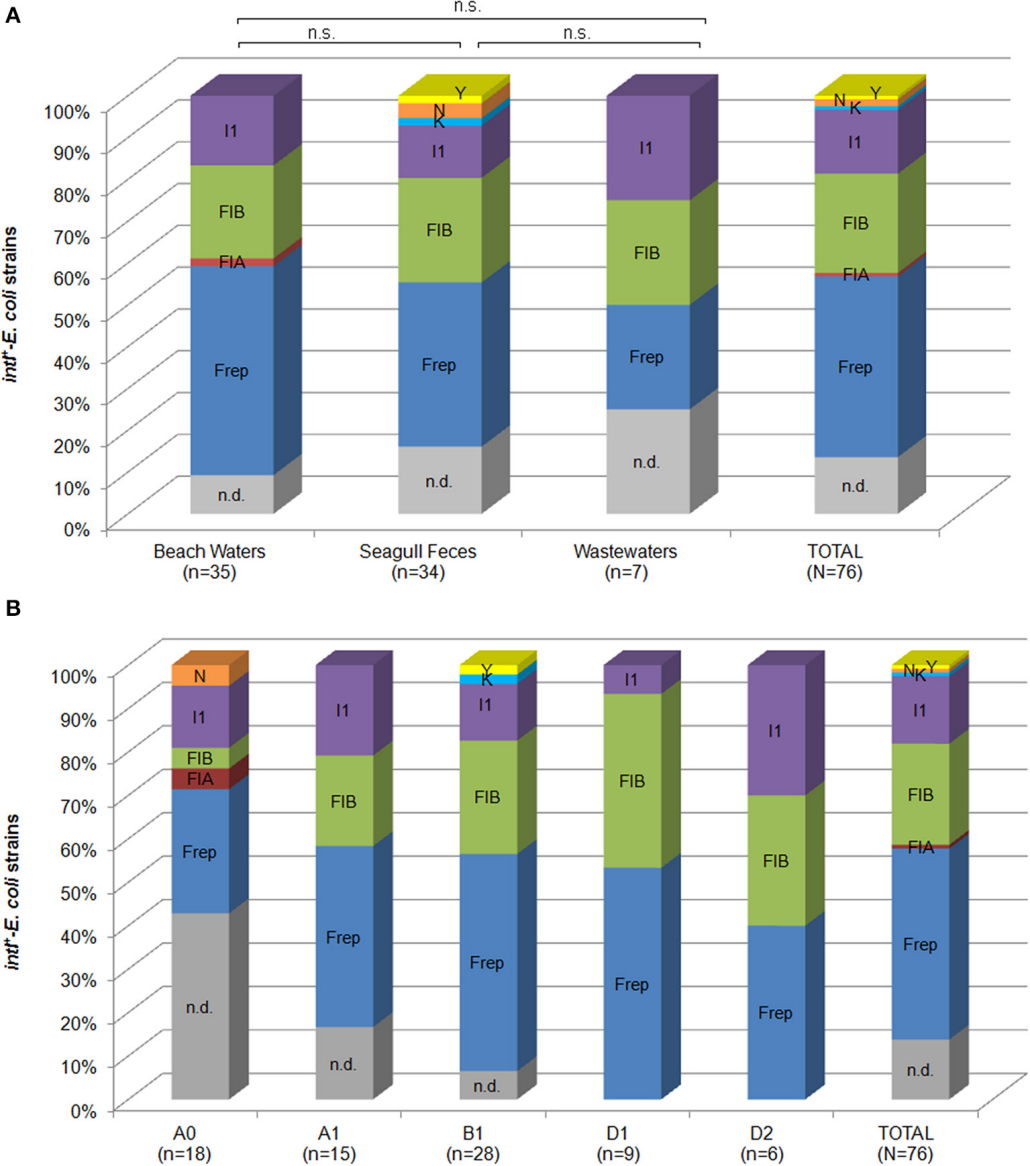
**Prevalence of replicon types detected in *intI^+^-E. coli* among different sources (A) and phylogroups (B)**. Abbreviations: n.d., not detected; n.s., not statistically significant.

Similar to previous reports on plasmid diversity among *intI*^+^-strains (Moura et al., 2012a), a wide and diverse plasmid pool was present in these *E. coli* (Figure 3A). Replicons were detected in 80% (60/76) of strains (Figure 3A; Table 2), though differences among BW, SF, and WW were not statistically significant. Replicons detected belonged to IncF (Frep, FIA, and FIB subgroups), IncI1, IncN, IncY, and IncK incompatibility groups. More than one replicon type was detected in 41% (31/76) of strains. In strains from phylogroups A0 and B1, up to 5 different replicon types were detected (Figure 3B). Integrons were successfully mobilized through IncF (Frep and FIB subgroups) and IncI1 conjugative plasmids into *E. coli* CV601 in 20% (15/76) of strains, using streptomycin as selective marker. The majority of *intI*-transconjugants displayed the resistance patterns observed in donor strains (Table 2), highlighting the importance of co-selection in the spread of multi-resistance traits through horizontal gene transfer. Plasmid DNA from transconjugants showed different restriction patterns (data not shown), including in transconjugants from donors that shared identical integron structures. These results may be explained by the presence of identical integron platforms in different plasmids. Nevertheless, the co-mobilization of multiple plasmids and/or the occurrence of genetic rearrangements in transconjugants resulting in different restriction patterns cannot be excluded. It is also noteworthy that plasmid prevalence and diversity, as well as their transfer ability may be, however, under-estimated due to biases introduced by the technical approaches. Alkaline extraction of plasmid DNA may affect the efficiency to recover larger plasmids, and the mating conditions used may favor the transfer the plasmids of IncF and IncI complexes, which are liquid maters.

In conclusion, results obtained confirmed the existence of a diverse integron pool in this coastal environment, associated with different resistance traits and plasmid incompatibility groups. The prevalence and diversity of integrons, as well as of multi-drug resistance phenotypes, found in beach waters were more influenced by animal-derived fecal inputs rather human-derived wastewaters. Results obtained thus reinforce the important input of commensal *E. coli* from wild animals in this ecosystem, largely dominated by seagulls. These findings underscore the role of wild life in dissemination of integrons and antibiotic resistance traits in natural environments.

### Conflict of interest statement

The authors declare that the research was conducted in the absence of any commercial or financial relationships that could be construed as a potential conflict of interest.
